# Supplementary material for: Region-Specific Gene Expression Changes Associated with Oleoylethanolamide-Induced Attenuation of Alcohol Self-Administration
Source: Int J Mol Sci. 2024 Aug 19;25(16):9002. doi: 10.3390/ijms25169002 (PMC11354326; doi:10.3390/ijms25169002)
Supplement: Supplementary file 1 [file ijms-25-09002-s001.zip › ijms-3117548-supplementary.pdf]

## SUPPLEMENTARY MATERIAL

**Table S1.** Assay codes of the primers used for the RT-PCR of genes.

| Gene | Assay codes |
|---|---|
| **Drd1** | Mm02620146 |
| **Drd2** | Mm00438545 |
| **Cnr1** | Mm01212171 |
| **Oprm1** | Mm01188089 |
| **β-Glucuronidase** | Mm00446953 |

**Table S2.** Nucleotide sequences of the primers used for the RT-PCR of genes.

| Gene | Primer sequences (5' to 3') |
|---|---|
| **Il-1β** | F: GACCCCAAAAGATGAAGGGCT<br>R: TGTGCTGCTGCGAGATTTGA |
| **Il-6** | F: AAGCCAGAGTCCTTCAGAGAGA<br>R: TCTTGGTCCTTAGCCACTCCT |
| **Tlr4** | F: TGCCTCTCTTGCATCTGGCTGG<br>R: CTGTCAGTACCAAGGTTGAGAGCTGG |
| **Bdnf** | F: CGCCAAGGTGGATGAGAGTT<br>R: TTCGGCTTTGCTCAGTGGAT |
| **Cyclophilin A** | F: GTCTCCTTCGAGCTGTTTGC<br>R: GATGCCAGGACCTGTATGCT |
